# Expression of Concern: Economic policy uncertainty and stock market in G7 Countries: A panel threshold effect perspective

**DOI:** 10.1371/journal.pone.0333748

**Published:** 2025-10-03

**Authors:** The PLOS One Editors

After this article [1] was published, concerns were noted about the article’s peer review and compliance with the PLOS Authorship policy.

In light of these concerns, the *PLOS One* Editors issue this Expression of Concern. Readers are advised to interpret the article [1] with caution.
