# Supplementary material for: Economic policy uncertainty and stock market in G7 Countries: A panel threshold effect perspective
Source: PLoS One. 2023 Jul 21;18(7):e0288883. doi: 10.1371/journal.pone.0288883 (PMC10361491; doi:10.1371/journal.pone.0288883)
Supplement: Attachment — Submitted filename: Respose to reviewrs.docx [file pone.0288883.s001.docx]

Review Comments to the Author

Reviewer #1: Line 48: Reiger and Wang
Line 54: Check reference criteria for direct quotations.

Clearly stipulate the Gap and contribution of the study.

S&P abbreviation?

Line 172 and onwards: Reference font is not consistent with the rest
Theoretical framework diagram can use different colour combinations for better clarity

Footnote 1: font is not consistent with the rest
Line 303 and 308: Avoid using bold in the text unless permitted by the journal format criteria

Line 350: Reference (and font) to be alligned with journal format requirement.

Response:

Dear anonymous referee, thanks for your time to review this article. We aggregated them to responded below. The revision is highlighted in green in revised manuscript, and brief responses are provided here.

The journal follows numbered citation/reference style, and we used EndNote that automatically formats all citations/references. We have converted most of the intext citations to indirect quotation that is consistent now throughout the manuscript, and fixed the direct quotation for remaining.

The gap/motivation of the study is articulated well in the revised manuscript (Introduction L29-39, Literature review L141-150).

S&P stands for Standard and Poor’s, and it is defined in revised manuscript.

Aligned with your recommendations color combination of theoretical diagram is adjusted.

The font is unified throughout the document (except for Headings, and tables).

Reviewer #2: I would like to thank the authors for compiling this article entitled; "Economic Policy Uncertainty and Stock Market in G7 Countries: A Panel Threshold Effect". At presently there are some elements in the paper. Please find the comments valued towards reshaping the paper. As of currently this research needs to be relooked. My comments towards the paper are as follows:

Response:

Dear anonymous referee, thanks for your time to review this article. We addressed all of your comments. The revision is highlighted in green in revised manuscript, and brief point to point responses are provided below.

1.In the abstract the aim of the study is highlighted but missing in the introduction part.

Response:

The aim of the study is added to introduction part as well.
2.There is missing link and justification of why it was important to carry out a study based on G7 countries.

Response:
we add following passage as justification on using the sample of G7 countries in introduction section:

*The G7 countries, including Canada, France, Germany, Italy, Japan, the United Kingdom, and the United States, are major global economies with substantial financial markets. These countries play a significant role in shaping global economic policies and have a substantial impact on international financial markets. Findings derived from studying the G7 countries have broader implications due to their global economic significance. As these countries are influential players in the international financial system, their financial market dynamics and policy implications can often be generalized to other countries or regions. Consequently, empirical research conducted on G7 countries can provide insights that are relevant and applicable to a wider range of economies and financial markets. Therefore, the use of the G7 countries as a sample in empirical research within the finance field is driven by their economic significance, diverse policy frameworks, availability of data, generalizability of findings, benchmarking value, and policy relevance.*

3. The first part of the introduction is aligned with the theoretical aspect of the study. I strongly suggest to move the theoretical underpinning shifted after the motivation of the paper.

Response:

Aligned with your recommendation theoretical part is moved after motivation, and motivation of the study is improved further (see first two paragraphs of introduction section).
4.It is important to first highlight the motivation of the paper and its distinctiveness .
5. Justify your claims of the findings with similar studies.

Response:

The motivation is highlighted in first para of introduction, and supporting studies are referred in last para of discussion section.
6.Literature review needs to be rearranged.
perhaps the authors might include a table for country specific studies and discuss the findings. in the table use heading such as Reference ,Country, Period, Variables, Method(s) and Findings. Use this study https://doi.org/10.1080/10941665.2020.1862884 a means to improve the literature and reference the table.
7. The literature seems to be basic. I would suggest to add themes in the literature. themes that are related to the study.

Response:

Literature review is rearranged and specifically a table is incorporated as per your suggestion taking theme form suggested research article (please see green highlighted text in literature section including Table 1).

8. There are different types of fonts used in the study. I suggest that it should be consistent.
Response:

The font is unified throughout the document (except for Headings, and tables).

I wish the authors all the best in doing the revision of the paper.

We would like to express our gratitude for your suggestion to present the major literature in a tabular format. This approach has been immensely helpful in providing us with a comprehensive overview of the dynamics between EPU and stock markets, allowing us to identify and articulate the existing gaps in the literature more effectively. Your input has been instrumental in shaping our understanding of the research landscape, and we sincerely appreciate your valuable contribution.
