# Supplementary material for: Economic policy uncertainty and stock market in G7 Countries: A panel threshold effect perspective
Source: PLoS One. 2023 Jul 21;18(7):e0288883. doi: 10.1371/journal.pone.0288883 (PMC10361491; doi:10.1371/journal.pone.0288883)
Supplement: Attachment — Submitted filename: Rebuttal letter.docx [file pone.0288883.s002.docx]

Review Comments to the Author

Reviewer #1: All comments and suggestions have been addressed. Please ensure that article meets all other journal formatting requirements. All the best with the article.

Response:

We extend regards to anonymous reviewer for encouraging remarks and accepting our response to the comments. We will consider formatting requirements of the journal.

Reviewer #2: I would like to thank the authors for providing valuable feedback towards the comments. This paper looks in shape from the previous version. I would still like point out some issues with the paper that still needs to be addressed.

Firstly my comment on adding themes in the literature. The authors can add separate section in the literature review and discuss the relevance of certain theories used in the study.
I also like to thank the authors in inserting a table in the literature. This has certainly added significance towards the paper.
The authors can also reference the article where the table is adopted from.
I wish the authors all the best in revising the paper.

Response:

We extend regards to anonymous reviewer for encouraging remarks and accepting our response to the comments. We address your comments which may be seen in green heighted text in revised manuscript, and brief reference is made below:

Following sentence is added on Page no 4, L#139-140:

“The theme of tabular presentation of core literature is adopted from Kumar, Chandra [1].”

Relevant theories to this study are briefly discussed in section created in literature section “**2.1** **Theoretical Underpinning**” as per your suggestion. Please see pp 4-5, L 114-174.

References

1.	Kumar, N.N., R.A. Chandra, and A. Patel, *Mixed frequency evidence of the tourism growth relationship in small Island developing states: a case study of Tonga.* Asia Pacific Journal of Tourism Research, 2021. **26**(3): p. 294-307.
